# Is informed consent related to success in exercise and diet intervention as evaluated at 12 months? DR's EXTRA study

**DOI:** 10.1186/1472-6939-11-9

**Published:** 2010-06-08

**Authors:** Helena Länsimies-Antikainen, Anna-Maija Pietilä, Tomi Laitinen, Vesa Kiviniemi, Rainer Rauramaa

**Affiliations:** 1 Department of Nursing Science, University of Eastern Finland, Yliopistonranta 1, 70211 Kuopio, Finland; 2 Unit of Clinical Physiology and Nuclear Medicine, Kuopio University Hospital, Puijonlaaksontie 2, 70210 Kuopio, Finland; 3 Institute of Clinical Medicine, Unit of Clinical Physiology and Nuclear Medicine, University of Eastern Finland, Yliopistonranta 1, 70211 Kuopio, Finland; 4 Information Technology Centre, University of Eastern Finland, Yliopistonranta 1, 70211 Kuopio, Finland; 5 Kuopio Research Institute of Exercise Medicine, Haapaniementie 16, 70100 Kuopio, Finland

## Abstract

**Background:**

There is a permanent need to evaluate and develop the ethical quality of scientific research and to widen knowledge about the effects of ethical issues. Therefore we evaluated whether informed consent is related to implementation and success in a lifestyle intervention study with older research participants. There is little empirical research into this topic.

**Methods:**

The subjects (n = 597) are a subgroup of a random population sample of 1410 men and women aged 57-78 years who are participating in a 4-year randomized controlled intervention trial on the effects of physical exercise and diet on atherosclerosis, endothelial function and cognition. Data were collected in two steps: A questionnaire about informed consent was given to all willing participants (n = 1324) three months after the randomization. Data on implementation and success in the exercise and diet interventions were evaluated at 12 months by intervention-group personnel. The main purpose of the analysis procedure performed in this study was to identify and examine potential correlates for the chosen dependent variables and to generate future hypotheses for testing and confirming the independent determinants for implementation and success. The nature of the analysis protocol is exploratory at this stage.

**Results:**

About half of the participants (54%) had achieved good results in the intervention. Nearly half of the participants (47%) had added to or improved their own activity in some sector of exercise or diet. Significant associations were found between performance in the interventions and participants' knowledge of the purpose of the study (p < 0.001), and between success in interventions and working status (p = 0.02), and the participants' knowledge of the purpose of the study (p = 0.04).

**Conclusion:**

The main finding of this study was that those participants who were most aware or had understood the purpose of the study at an early stage had also attained better results at their 12-month intervention evaluation. Therefore, implementation and success in intervention is related to whether subjects receive a sufficient amount and are able to comprehend the information provided i.e. the core principles of informed consent.

**Trial Registration:**

(ISRCTN 45977199)

## Background

It is often stated that prevention is better than cure and thus lifestyle interventions are important topics for research. For example, the number of individuals with diabetes is growing at an alarming rate. Prompt intervention by promoting and facilitating improvements in diet, activity levels, and body weight will not only result in prevention of diabetes, but also achieve overall improvements in physical and mental health [1]. However, these kinds of studies are not possible without the participation of willing human subjects [2-4]. The participants' trust and interest in scientific research can be maintained and their enthusiasm to take part in clinical trials can be promoted by ensuring that the research trial adheres to high ethical standards. Therefore, there is a clear need to evaluate and develop the ethical quality of research and to widen our knowledge about the impacts of different ethical aspects. One such aspect is clarifying how informed consent can influence the success of participants in these studies, especially with older research participants.

Studies have been conducted into the effectiveness of health promotion interventions, for example, research into how the elderly respond to exercise or physical activity [5-7]. However, there is little empirical research about the success related to informed consent or commitment to intervention. In addition, issues related to the positive or negative utility associated with participating in a preventive health programme are often ignored [8]. The objective of this short report was to evaluate whether informed consent would be related to the implementation and success in an exercise and diet intervention study, as evaluated after 12 months of intervention. The crucial question was: Does the participant's satisfaction with the informed consent process in the early stages improve implementation and success of intervention in health research? In addition, we were interested to elucidate whether the participant's background variables were related to implementation and success in this kind of lifestyle intervention study.

## Methods

### Participants and Design of the study

The participants (n = 597) included here are part of a study group consisting of 1410 men and women aged 57-78 years who are participating in a randomized controlled intervention trial on the effects of regular physical exercise and diet (DR's EXTRA study: Dose-Responses to Exercise Training. A randomized controlled trial on the effects of regular physical exercise and diet on endothelial function, atherosclerosis and cognition). In 2002, a representative 15% sample (n = 3000) of all 55- to 74-year-old men and women living in the city of Kuopio (Finland) was invited to participate in an exercise and diet intervention study. Of the subjects invited initially, 2062 expressed an interest in participating and 1410 subjects participated in all four baseline examinations conducted between April 2005 and November 2006. The main exclusion criteria at entrance were conditions that would inhibit safe engagement in the prescribed exercise training, malignant diseases as well as other conditions preventing potential participants from co-operating, as judged by the research physicians.

**Figure 1:**
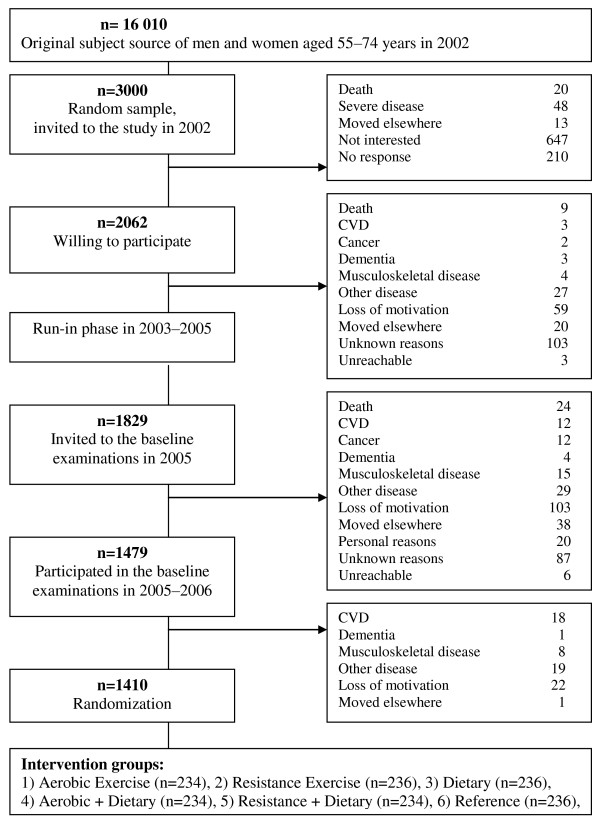
**Study design at DR's EXTRA study**.

The participants were randomized into six intervention groups: 1) Reference, 2) Aerobic Exercise, 3) Resistance Exercise, 4) Diet, 5) Aerobic Exercise and Diet, 6) Resistance Exercise and Diet. The design of the DR's EXTRA study is presented in detail in Figure 1. Both the exercise and diet intervention study and the 'Informed Consent' study were approved by the Research Ethics Committee of the Hospital District of Northern Savo and written informed consent was obtained.

The ***aerobic exercise group ***was provided with an individualized training program performed at an intensity of 55-65% of maximal oxygen uptake. Training frequency, duration and intensity were gradually increased during the first six months. Thereafter, the subjects were allocated into two subgroups of different weekly exercise doses: 1000-1500 kcal/wk (60 min, 5 times/wk) or > 1500 kcal/wk (90 min, 5 times/wk).

In the ***resistance exercise group***, the subjects participated in supervised, individually prescribed strength training programs. Training frequency, duration and intensity were gradually increased during the first six months. Thereafter, the subjects were randomized into two subgroups receiving different weekly exercise doses: 1000-1500 kcal/wk (2 session/wk) or > 1500 kcal/wk (3 sessions/wk). In each session, 10-14 muscle groups were trained at an intensity of 60% of maximum, 2 sets, 15 repetitions/set. In addition, the subjects were advised to undertake aerobic exercise twice a week.

In the ***diet group***, the participants received counselling by nutritionists. During the first six months, all participants were provided with instructions based on Finnish Nutrition Recommendations (FNR). The main goals were to substitute unsaturated for saturated fat and to increase the intake of fibre and antioxidants. After six months, the subjects were randomly allocated into two subgroups: FNR or Special Nutrition (SN) group. The FNR group continued with the same recommendations. The SN group was given additional instructions: to increase their use of vegetables, fruits, berries, chicken, nuts and almonds as well as decreasing their consumption of red meat.

### Data collection

First, data on the participant's opinion about the informed consent process were collected by questionnaire over a 23-month period in 2005-2007. The questionnaire was self-administered and tested in a pilot study [9]. The key elements of informed consent were defined as follows: information, understanding, competence, voluntariness, and decision-making. During the three-month intervention visit at Research Institute of Exercise Medicine, the questionnaire was given to all willing participants (n = 1324) who were still involved in the study. In the 'informed consent' study, 1200 participants returned the questionnaire, the response rate being 91%.

**Figure 2:**
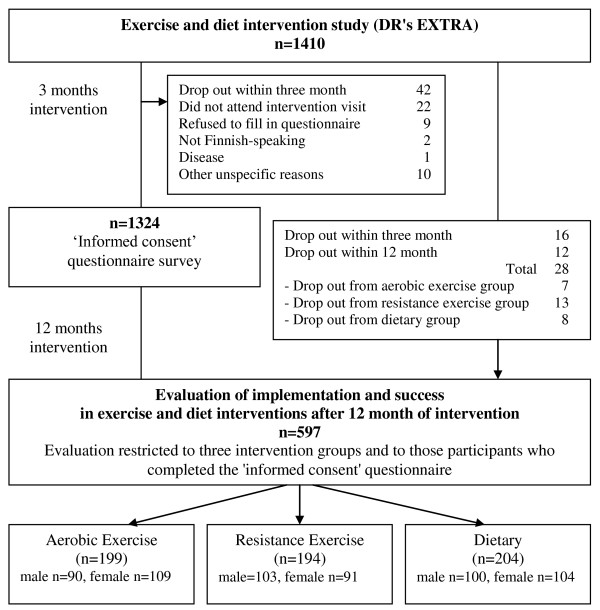
**Study design in this study**.

Second, the data on implementation and success in exercise and diet intervention study after 12 months of intervention were measured by the trial personnel. They evaluated the following two questions: 1) How has the intervention been implemented in the exercise and diet intervention study after 12 months of intervention? 2) How successful have the participants been in the study after 12 months' intervention, as measured by changes in their activities with respect to exercise or diet? The design of this study is presented in detail in Figure 2.

### Data analysis

In the data analysis, we took into account three intervention groups (aerobic exercise, resistance exercise, and diet) and omitted the other three groups. The reason for this selection was that in the reference group the participants received no intervention, and in the combined exercise and diet groups, two members of the intervention-group personnel evaluated the participant's success in each intervention separately. In addition, the data analysis was restricted to those participants who completed the 'informed consent' questionnaire. As a consequence, the number of participants totalled 597. From the 'informed consent' questionnaire, we selected those questions which were relevant to the research questions addressed in this paper. The list of variables examined in all analyses and their classifications are presented in Table 1.

**Table 1:** List of variables used in data analyses and their codes and classifications

List of variables	Codes and Classification
**Response variables**	

	

Result of the intervention	1) attained good result (over 70%), 2) attained moderate result (31-69%), 3) attained poor result (0-30%)

The success of an intervention measured by change in activity	1) added/improved in many sectors, 2) added/improved in some sectors, 3) no change, 4) reduced/worsened

	

**Background variables**	

	

Gender	1) male, 2) female*

Age	< 63, 64-69, > 70 years*

Marital status	1) married, 2) unmarried*

Education	1) no professional training, 2) vocational school or vocational course, 3) college-level training, 4) academic degree*

Work status	1) working, 2) not working*

Own opinion of own health	1) poor or extremely poor, 2) moderate, 3) good, 4) extremely good*

Earlier participation in research projects	1) yes, 2) no*

	

**Other variables**	

	

Participant's knowledge of person in charge	1) yes, 2) no*

Participant's engagement with contact person	1) yes, 2) no, 3) had no need to contact*

Opinion of sufficiency of time during the first visit	0) poor to moderate, 1) good*

Opinion of sufficiency of information given	0) poor to moderate, 1) good*

Opinion of intelligibility of information given	0) poor to moderate, 1) good*

Opinion of sufficiency of information about participants' selection criteria to the study	0) poor to moderate, 1) good*

Adequate possibility to consider participation	1) yes, 2) no*

Participant's view of the purpose of the study	1) answered correctly*, 0) answered incorrectly or left empty

Opinion of whether the research personnel had adequately confirmed that the participant received enough information	0) poor to moderate, 1) good*

Opinion of whether the research personnel had adequately confirmed that the participant had understood the information given	0) poor to moderate, 1) good*

* Referent categories

Of these 597 participants 10 dropped out before the 12 months' intervention. These being four from the aerobic exercise group, three from the resistance exercise group, and three from the diet group. The drop out reasons were as follows: death (n = 2), disease (n = 2), moved elsewhere (n = 1), loss of motivation (n = 1), personal reasons (n = 3), unknown reason (n = 1). The details of the drop outs have been compared with those of the participants still in the study. This comparison (cross-tabulations and Fisher's Exact Test) did not detect any statistically significant difference between baseline characteristics (background variables or intervention group). In addition, the differences that were checked between the drop outs and those who did not drop out were considered not to be clinically important.

Evaluations of implementation and success in the exercise and diet interventions were based on the participants' records and supplemented by intervention-group personnel's subjective estimation. For example, in the aerobic exercise group, the participants recorded the amount, duration and type of their training. In the resistance exercise group, the training record was stored in a personal smart card kept at the fitness facility of the institute, and in the diet group, the participants filled in a food diary. The bookkeeping was scored by a predetermined grading system. In addition, the intervention-group personnel met the participants regularly and interviewed and in this way, they ensured that the subjective evaluation was reliable.

The data were analyzed using the SPSS for Windows software (v. 14.0, 2005, SPSS Inc., Chicago, IL). First, frequencies and percentages were calculated to describe the data. Second, univariate analyses were calculated for every variable examined. (See the Additional file 1: Univariate data analyses.) In the multivariable analyses, those variables were chosen which had a p-value of ≤ 0.1 in the univariate analyses. Third, backward directed stepwise multivariable analyses were used to identify correlates of variables concerning implementation and success. In the multivariable analyses, regression models were used taking p < 0.05 to identify significant predictors of outcomes. P-values from all steps, and p-values, estimates, and standard errors are reported from the last steps of the multivariable regression models. Only the models including main effects were fitted. Interactions (effect modifications) were not examined.

The main purpose of the analysis procedure performed in this study was to identify and examine the potential correlates for the chosen dependent variables and to generate future hypotheses for testing and confirming the independent determinants for implementation and success. The nature of the analysis protocol is exploratory at this stage.

## Results

### Background information

The age of the participants (293 male and 304 female) varied from 57 to 78 years (mean 67 years, SD 5 years). The majority of the participants (75%) were married or living in a common-law relationship. The participants' educational level was as follows: 37% had vocational school or equivalent background, 26% had college-level training, 22% had no professional training, and 16% had an academic degree. The vast majority of the participants (87%) were retired, and the rest (13%) were working either full- or part-time. A minority of the participants (37%) had previously participated in a research project. More than half (56%) were of the opinion that their health was moderately good and 37% felt that their health was good. Four percent felt that their health was extremely good and 3% stated it was poor or extremely poor.

### Implementation of the intervention

About half of the participants (54%) were estimated to have attained good results in the interventions. About one-third of the participants (35%) had attained a moderate result and a minority of the participants (12%) were considered as having a poor result. Statistically significant associations were found between the implementation of interventions and the participants' knowledge of the purpose of the study (p < 0.001). Those participants who were most aware or had understood the purpose of the study had also attained better results in their intervention when it was evaluated after 12 months. (See Table 2.)

**Table 2:** Implementation of intervention at the exercise and diet intervention study

Variables	Step 1 highest p-values	Step 2 highest p-values	Step 3 highest p-values	**Last step **p-value	**Last step **Estimate (location)	Last step Std Error
Personal opinion of subject's own health	0.7	drop	-	-		

Marital status	0.33	0.17	drop	-		

Confirmation that the research personnel had ensured that the participant received enough information	0.23	0.11	0.09	drop		

Participant's view of the purpose of the study	0.001	<0.001	<0.001	<0.001		
- answered incorrectly					0.49	0.13
- answered correctly					0	

P-values from all steps, and p-values, estimates, and standard errors of the most predictive variables selected by the stepwise procedure (step 4) of the ordinal regression model.

### Success of the intervention

Nearly half of the participants (47%) had added to or improved personal involvement in some sector of exercise or diet. Almost one-fifth of the participants (18%) had supplemented to or improved their own involvement in many sectors of exercise or diet. A third of the participants (33%) undertook no changes in their activity in terms of exercise or diet; a few of the participants (1%) had reduced or worsened activity. There were statistically significant associations between success in interventions and 1) working status (p = 0.02) and 2) participants' knowledge of the purpose of the study (p = 0.04). Participants who were still in employment or who were most aware or had understood the purpose of the study had also succeeded better in their intervention, as revealed at the 12 month evaluation. (See Table 3.)

**Table 3:** Success of the intervention measured by the change in activity during the exercise and diet intervention study

Variables	Step 1 highest p-values	Step 2 highest p-values	Last step p-values	**Last step **Estimate (location)	Last step Std Error
Personal opinion of subject's own health	0.64	drop	-		

Marital status	0.12	0.08	drop		

Working status	0.01	0.03	0.02		
- working				-0.33	0.14
- not working				0	

Participant's view of the purpose of the study	0.07	0.06	0.04		
- answered incorrectly				0.26	0.13
- answered correctly				0	

P-values from all steps, and p-values, estimates, and standard errors of the most predictive variables selected by the stepwise procedure (step 3) of the ordinal regression model.

## Discussion

The main finding of this study was that both the implementation of the intervention and the participants' success in the intervention were related to their knowledge of the purpose of the study in question. In other words, those participants who were most aware or had understood the purpose of the study had also attained better results in the lifestyle intervention, at the 12 month evaluation.

The main strength of this study is that it is based on a population-based intervention study in which a large number of men and women are participating. In addition, the response rate was very high. The major limitations were the restricted duration of the intervention and that the inter-rater reliability and concurrent validity were not evaluated.

The open question about the purpose of the study was asked to evaluate the respondents' ability to understand information that had been provided. These replies supported the belief that the information had been understood appropriately by a majority of the study group, i.e. most of the respondents (82%) answered correctly. Though there is more regulatory scrutiny of consent forms, there are still known to be deficiencies in participants' comprehension of the research in which they participate. There are also differences in the ways how comprehension is measured and assessed [10]. In addition, it is very difficult to estimate how well a participant has understood the information received [11]. The need to ensure adequate comprehension is inseparable from the requirement that information be disclosed [12]. In addition, in one study, the reported side effects mirrored the information provided to participants at the time when informed consent was given [13]. Here we have extended this concept to participants' success in the interventions. These findings highlight the need for researchers to ensure that participants have received sufficient relevant information and that they have understood it [14]. The investments involved and effort exerted not only affect the participants' success and commitment to the research in question, but also can impact on the reliability of the research result. Therefore, all researchers must critically analyze the quality and stability of information as well as determining how it has been delivered in their study.

## Conclusion

The main purpose of the analysis procedure performed in this study was to identify and examine the potential correlates for the chosen dependent variables and to generate future hypotheses for testing to confirm these independent determinants for implementation and success. The implementation and success in intervention seem to be related to the sufficiency and comprehension of information received. One important fact is that both information and its comprehension are major elements of informed consent. Thus, if one asks the question "Is informed consent related to success in an exercise and diet intervention?" the answer is yes.

## Competing interests

The authors declare that they have no competing interests.

## Authors' contributions

HL-A, A-MP, TL and RR were responsible for the conception and design of the study. HL-A performed or co-ordinated the data collection, data analysis and drafting of the manuscript. VK gave support and made critical revisions to the data analysis. A-MP, TL and RR critically revised the manuscript. RR provided administrative support and supervision. All authors read and approved the final manuscript.

## Pre-publication history

The pre-publication history for this paper can be accessed here:

http://www.biomedcentral.com/1472-6939/11/9/prepub

## Supplementary Material

Additional file 1 **Univariate data analyses**. This data includes two tables. Table 1 presents the results of univariate analyses when evaluated the implementation of intervention at the exercise and diet intervention study. Table 2 presents the results of univariate analyses when evaluated the success of the intervention was evaluated by the change in activity during the exercise and diet intervention study. Click here for file
