# Supplementary material for: Is informed consent related to success in exercise and diet intervention as evaluated at 12 months? DR's EXTRA study
Source: BMC Med Ethics. 2010 Jun 8;11:9. doi: 10.1186/1472-6939-11-9 (PMC2891796; doi:10.1186/1472-6939-11-9)
Supplement: Additional file 1 — Univariate data analyses. This data includes two tables. Table 1 presents the results of univariate analyses when evaluated the implementation of intervention at the exercise and diet intervention study. Table 2 presents the results of univariate analyses when evaluated the success of the intervention was evaluated by the change in activity during the exercise and diet intervention study. [file 1472-6939-11-9-S1.DOC]

**Additional file 1:** Univariate data analyses

**Table 1** Results of univariate analyses when evaluated the implementation of intervention at the exercise and diet intervention study.

| **Variables** | **Classification** | **estimate**  **(location)** | **std error** | **p-value** (mod.fitt) | **p-values** |
| --- | --- | --- | --- | --- | --- |
| Gender | male  female | -0.02  0 | 0.09 | 0.8 | 0.8 |
| Age | < 63 years  64-69 years  > 70 years | -0.02  -0.14  0 | 0.12  0.11 | 0.4 | 0.89  0.22 |
| Marital status | married  unmarried | -0.2  0 | 0.11 | **0.06** | 0.07 |
| Education | no professional training  vocational school or course  college-level training  academic degree | 0.04  -0.05  -0.15  0 | 0.15  0.14  0.15 | 0.53 | 0.8  0.75  0.32 |
| Work status | working  not working | 0.05  0 | 0.14 | 0.72 | 0.72 |
| Own opinion of own health | poor or extremely poor  moderate  good  extremely good | 0.99  0.23  -0.3  0 | 0.4  0.23  0.24 | **<0.001** | 0.01  0.32  0.20 |
| Earlier participation in research projects | yes  no | 0.01  0 | 0.1 | 0.92 | 0.92 |
| Participant's knowledge of person in charge | yes  no | 0.03  0 | 0.11 | 0.8 | 0.8 |
| Participant's engagement with contact person | yes  no  had no need to contact | 0.13  0.02  0 | 0.1  0.4 | 0.43 | 0.2  0.97 |
| Opinion of sufficiency of time during the first visit | poor or moderate  good | 0.06  0 | 0.21 | 0.77 | 0.78 |
| Opinion of suffuciency of information given | poor to moderate  good | 0.1  0 | 0.14 | 0.46 | 0.47 |
| Opinion of intelligibility of information given | poor or moderate  good | 0.14  0 | 0.16 | 0.34 | 0.35 |
| Opinion of sufficiency of information about participants’ selection criteria to the study | poor or moderate  good | 0.003  0 | 0.09 | 0.98 | 0.98 |
| Adequate possibility to consider participation | yes  no | -0.66  0 | 0.55 | 0.16 | 0.23 |
| Participant’s view of the purpose of the study | answered correctly  answered incorrectly or left empty | 0.49  0 | 0.13 | **<0.001** | <0.001 |
| Opinion about whether the research personnel had adequately confirmed that the participant received enough information | poor or moderate  good | 0.18  0 | 0.1 | **0.07** | 0.08 |
| Opinion about whether the research personnel had adequately confirmed that the participant had understood the information given | poor or moderate  good | 0.12  0 | 0.1 | 0.21 | 0.21 |

**Table 2** Results of univariate analyses when evaluated the success of the intervention measured by the change in activity during the exercise and diet intervention study.

| **Variables** | **Classification** | **estimate**  **(location)** | **std error** | **p-value** (mod.fitt) | **p-values** |
| --- | --- | --- | --- | --- | --- |
| Gender | male  female | 0.02  0 | 0.09 | 0.82 | 0.82 |
| Age | < 63 years  64-69 years  > 70 years | -0.13  -0.01  0 | 0.12  0.11 | 0.45 | 0.26  0.9 |
| Marital status | married  unmarried | -0.24  0 | 0.11 | **0.03** | 0.03 |
| Education | no professional training  vocational school or course  college-level training  academic degree | 0.11  0.1  <0.001  0 | 0.16  0.14  0.15 | 0.77 | 0.5  0.49  1 |
| Work status | working  not working | -0.36  0 | 0.14 | **0.01** | 0.01 |
| Own opinion of own health | poor or extremely poor  moderate  good  extremely good | 0.32  -0.16  -0.41  0 | 0.36  0.23  0.24 | **0.009** | 0.38  0.5  0.09 |
| Earlier participation in research projects | yes  no | -0.02  0 | 0.1 | 0.84 | 0.84 |
| Participant's knowledge of person in charge | yes  no | 0.15  0 | 0.11 | 0.18 | 0.18 |
| Participant's engagement with contact person | yes  no  had no need to contact | 0.06  0.16  0 | 0.1  0.41 | 0.82 | 0.58  0.7 |
| Opinion of sufficiency of time during the first visit | poor or moderate  good | 0.01  0 | 0.21 | 0.98 | 0.98 |
| Opinion of suffuciency of information given | poor to moderate  good | 0.18  0 | 0.14 | 0.19 | 0.2 |
| Opinion of intelligibility of information given | poor or moderate  good | 0.03  0 | 0.15 | 0.84 | 0.84 |
| Opinion of sufficiency of information about participants’ selection criteria to the study | poor or moderate  good | 0.002  0 | 0.09 | 0.99 | 0.99 |
| Adequate possibility to consider participation | yes  no | -0.32  0 | 0.50 | 0.52 | 0.53 |
| Participant’s view of the purpose of the study | answered correctly  answered incorrectly or left empty | 0.29  0 | 0.13 | **0.02** | 0.02 |
| Opinion about whether the research personnel had adequately confirmed that the participant received enough information | poor or moderate  good | 0.05  0 | 0.10 | 0.58 | 0.59 |
| Opinion about whether the research personnel had adequately confirmed that the participant had understood the information given | poor or moderate  good | 0.02  0 | 0.1 | 0.82 | 0.82 |
